# Identifying and correcting spatial bias in opportunistic citizen science data for wild ungulates in Norway

**DOI:** 10.1002/ece3.8200

**Published:** 2021-10-05

**Authors:** Benjamin Cretois, Emily G. Simmonds, John D. C. Linnell, Bram van Moorter, Christer M. Rolandsen, Erling J. Solberg, Olav Strand, Vegard Gundersen, Ole Roer, Jan Ketil Rød

**Affiliations:** ^1^ Department of Geography Norwegian University of Science and Technology Trondheim Norway; ^2^ Norwegian Institute for Nature Research Trondheim Norway; ^3^ Department of Mathematical Sciences Norwegian University of Science and Technology Trondheim Norway; ^4^ Department of Forestry and Wildlife Management Inland Norway University of Applied Sciences Koppand Norway; ^5^ Faun Naturforvaltning AS Fyresdal Norway

**Keywords:** citizen science, habitat selection, opportunistic data, preferential sampling, spatial bias, ungulates

## Abstract

Many publications make use of opportunistic data, such as citizen science observation data, to infer large‐scale properties of species’ distributions. However, the few publications that use opportunistic citizen science data to study animal ecology at a habitat level do so without accounting for spatial biases in opportunistic records or using methods that are difficult to generalize. In this study, we explore the biases that exist in opportunistic observations and suggest an approach to correct for them. We first examined the extent of the biases in opportunistic citizen science observations of three wild ungulate species in Norway by comparing them to data from GPS telemetry. We then quantified the extent of the biases by specifying a model of the biases. From the bias model, we sampled available locations within the species’ home range. Along with opportunistic observations, we used the corrected availability locations to estimate a resource selection function (RSF). We tested this method with simulations and empirical datasets for the three species. We compared the results of our correction method to RSFs obtained using opportunistic observations without correction and to RSFs using GPS‐telemetry data. Finally, we compared habitat suitability maps obtained using each of these models. Opportunistic observations are more affected by human access and visibility than locations derived from GPS telemetry. This has consequences for drawing inferences about species’ ecology. Models naïvely using opportunistic observations in habitat‐use studies can result in spurious inferences. However, sampling availability locations based on the spatial biases in opportunistic data improves the estimation of the species’ RSFs and predicted habitat suitability maps in some cases. This study highlights the challenges and opportunities of using opportunistic observations in habitat‐use studies. While our method is not foolproof it is a first step toward unlocking the potential of opportunistic citizen science data for habitat‐use studies.

## Graphical abstract

We provide a novel method to use citizen science data for fine‐scale studies.

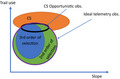

## 1 INTRODUCTION

Modern biotelemetry devices using very high frequency (VHF) and Global Positioning System (GPS) approaches have made it possible to study the habitat use of multiple animals at fine spatial and temporal scales, providing unique opportunities to study how species use their environment without observer bias (Frair et al., 2010). Nevertheless, such devices are expensive, often logistically difficult to deploy, and require specialist training in addition to the welfare considerations associated with animal capture. The result is that these approaches are often only used in study sites of limited size or with limited number of study animals, which may lead to poor population‐level inferences (Hebblewhite & Haydon, 2010) and cannot be applied to all species for which such information is desirable. Ideally, it should be possible to use available biotelemetry data, and correct for, biases associated with the use of more extensive data types (which are often opportunistic), such as those associated with citizen science data sources.

Opportunistic citizen science data have the potential to provide tremendous amounts of data over large temporal and spatial scales that can potentially transform the study of ecology (Bela et al., 2016; Tewksbury et al., 2014). It has recently been estimated that as much as 50% of the species occurrence records stored in the Global Biodiversity Information Facility (GBIF) have been collected by Citizen Scientists (i.e., volunteers engaged in data collection), usually in the form of opportunistic data (Walker, 2019). Many citizen science projects have a long history (e.g., hunters recording harvest numbers, Cretois et al., 2020; records of the timing of cherry blossom in Japan, Aono & Kazui, 2008; the UK Butterfly Monitoring Scheme, Mair et al., 2014; the Christmas Bird Count, Kobori et al., 2016), and the development of web‐based recording with user‐friendly interfaces and associated databases is leading to an increase in the number of initiatives and an increasing uptake by the scientific community (Dickinson et al., 2012). Where these datasets have a sufficient spatial and temporal resolution, they can represent a cost‐effective tool for certain applications such as delineating relative distributions or identifying habitat correlates for 1st‐order selection (Johnson, 1980). However, opportunistic data do not arise from any structured sampling design and thus violates many of the fundamental principles of data sampling. For instance, most data collected by volunteers are unevenly distributed in both space (i.e., off and on trails, close to roads, and human settlements, Westekemper et al., 2018) and time (i.e., collected during daylight and during the weekends) and might lead to spurious inference about drivers of species distribution. Moreover, observers differ in their abilities to recognize species and the effort they spend to detect certain species (Isaac et al., 2014). There is also a question whether the observed individuals are representative of the main wildlife population, or if they have deviant behavior, for example, because they are sick or more than usual habituated to human activities (Reimers et al., 2010). Even though at fine scales these biases can lead to misleading conclusions if not accounted for (Sicacha‐Parada et al., 2020), some studies chose to simply ignore them (Weisshaupt & Rodríguez‐Pérez, 2017), uncritically combine opportunistic records with other source of data (Mononen et al., 2018), or use methods difficult to replicate in other systems (Todd et al., 2016).

**FIGURE 1:**
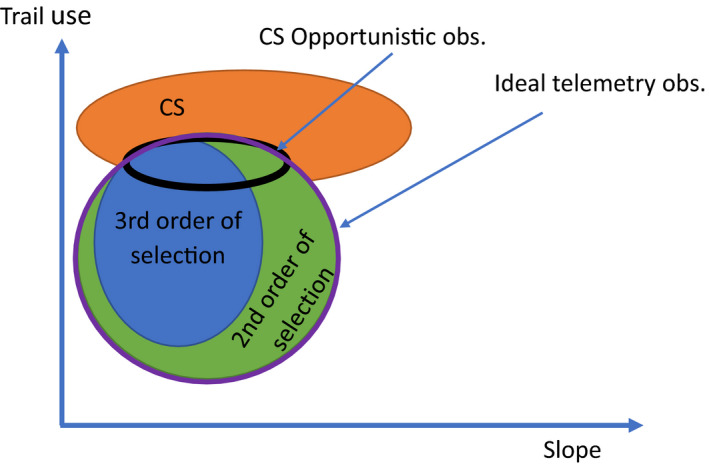
Conceptual figure representing the reasoning underlying the use of opportunistic observations to infer species’ habitat preference along two potential environmental gradients. The thick line represents the area where opportunistic observations correctly identify species’ ecological properties, including the RSF

It is commonly assumed that opportunistic data represent the actual species distribution. This is only partially true as opportunistic data represents the intersection between opportunistic sampling and the actual species distribution (see a two‐dimensional example in Figure 1). The environmental conditions determining occupancy by a species result from a hierarchical selection process (Johnson, 1980), while the fact that opportunistic data are conditional upon the presence of an observer and their ability to see and identify the animal, and file a report, are sources of bias. In contrast, the hypervolume in environmental space occupied by telemetry data results only from the space use of marked individuals from the species of interest. For instance, in Figure 1, citizen scientists and the target species do not use the landscape in the same way and citizen science observations only partially capture the 2nd and 3^rd^ orders of selection (Johnson, 1980). In contrast, ideal telemetry observations (i.e., exempt of sampling biases) are in theory able to capture both the 2nd and 3rd order of selection. In this example, citizen scientists use steeper slope and heavily used trails compared to the target species that prefer less steep landscapes that contains trails that are moderately used. Thus, the distribution of the citizen scientists and the species only partially overlap. Under the assumption of a representative sample of individuals in the telemetry data within a given site, it is possible to combine opportunistic and telemetry data to estimate the hypervolume occupied by the observers, which could be used to correct observer bias in the opportunistic data.

In this study, we present a novel method which aims to account for spatial biases in opportunistic observations to get a more accurate characterization of species’ habitat selection in an area rich in opportunistic data but where relatively little telemetry data are available. We build on previous studies which have found that carefully selecting background locations or using design‐based weights can help account for latent sampling bias and improve improves the inference made on species distribution (see Irvine et al., 2018; Phillips et al., 2009) and extend these ideas to a higher order of selection (Johnson, 1980). We first explore the potential biases in opportunistic observations for three widespread and easily recognizable wild ungulate species (i.e., to limit the extent of the misidentification bias), roe deer (*Capreolus capreolus*), moose (*Alces alces*), and wild mountain reindeer (*Rangifer tarandus*) in southern Norway. Then, we account for these biases by fitting a model aimed at estimating accessibility for the citizen scientist (the observer model) based on the contrast between observation and telemetry locations. We use the observer model output to define the spatial domain of a background sample. We then pair these background points with the opportunistic observations to estimate a resource selection function (RSF) that accounts for sampling bias. We further explored the potential of this method with both simulations and empirical datasets for the three wild ungulates. We compared the results of our novel method to RSFs naïvely using opportunistic observations without correction in availability locations and to RSFs derived from the unbiased telemetry data.

## 2 MATERIAL AND METHODS

### 2.1 Empirical data

#### 2.1.1 Telemetry data

We used GPS‐telemetry data collected between 2008 and 2017 from a total of 501 individuals (*n*_moose_ = 116, *n*_roe deer_ = 49, *n*_wild reindeer_ = 294) located in southern Norway (more details on data collection and study site locations in Roer et al., 2018 for moose; Peters et al., 2017 for roe deer, and Panzacchi et al., 2015 for wild reindeer). Because of the geographical particularities of the area in which the roe deer GPS collar data were located, we complemented the GPS dataset with VHF‐telemetry data from Viken and Innlandet counties in order to represent a wider diversity of landscape types. The VHF data were collected using either ground‐based triangulation or aerial locations. Even though VHF data are not as accurate as GPS telemetry, we chose to include them due to their wider coverage. The VHF data were obtained from 41 individuals and were collected between 1995 and 2004. Even though telemetry data have its own set of biases (e.g., capture locations are often conveniently placed for human access, some sort of bait or lure can be used to attract a certain individual), we carefully selected the telemetry data used in this study. Thus, we assume that telemetry data are, here, highly correlated to the real distribution of the species.

For all species, the GPS data sampling interval ranged between 1 and 12 relocations per day. However, because of the large number of data points, which caused computational inefficiency, and to avoid risks of temporal and spatial autocorrelation, we resampled the telemetry dataset using the R package amt (Signer et al., 2019). We selected 1 GPS location every 5 h for both moose and roe deer and 1 GPS location every 10 h for wild reindeer as more observations were available. We tested different filtering to ensure independence and we notice that from 5 (for roe deer and moose) and 10 (for wild reindeer) hours onward the parameter estimates remained stable. Then, we selected location data that were recorded during summer (i.e., from June 22 to September 22) and during hours of normal human activities (i.e., between 8 and 22) and daylight for a fair comparison with opportunistic observations, which are more numerous in the summer months (in our dataset) and during daylight. Focusing on summer only also removed complications arising from variable migration behavior and possible confounding effects of proximity to winter feeding stations that are often used by moose and roe deer, as well as issues related to the increased grouping behavior of moose and roe deer in winter and the reduced human access to habitats caused by snow (Fryxell et al., 1988).

#### 2.1.2 Opportunistic data

We extracted moose, roe deer, and wild reindeer records from the Norwegian Species Observation Service (https://www.artsobservasjoner.no/) dataset that we downloaded from GBIF (Norwegian Biodiversity Information Centre & Hoem 2021). “Artsobservasjoner” is the most popular citizen science platform in Norway and is maintained by the Norwegian Biodiversity Information Centre (https://www.artsdatabanken.no/). The system records individual species observations as point locations with no structure to its sampling protocol, such that data are purely opportunistic. We retained all observations that were recorded during the summer season for all years (ranging from 1990 to 2021, with more than 50% of observations being recorded between 2015 and 2021) and that had coordinate uncertainty of less than 400 m. The hour of observation was not recorded in the database.

**MAP 1:**
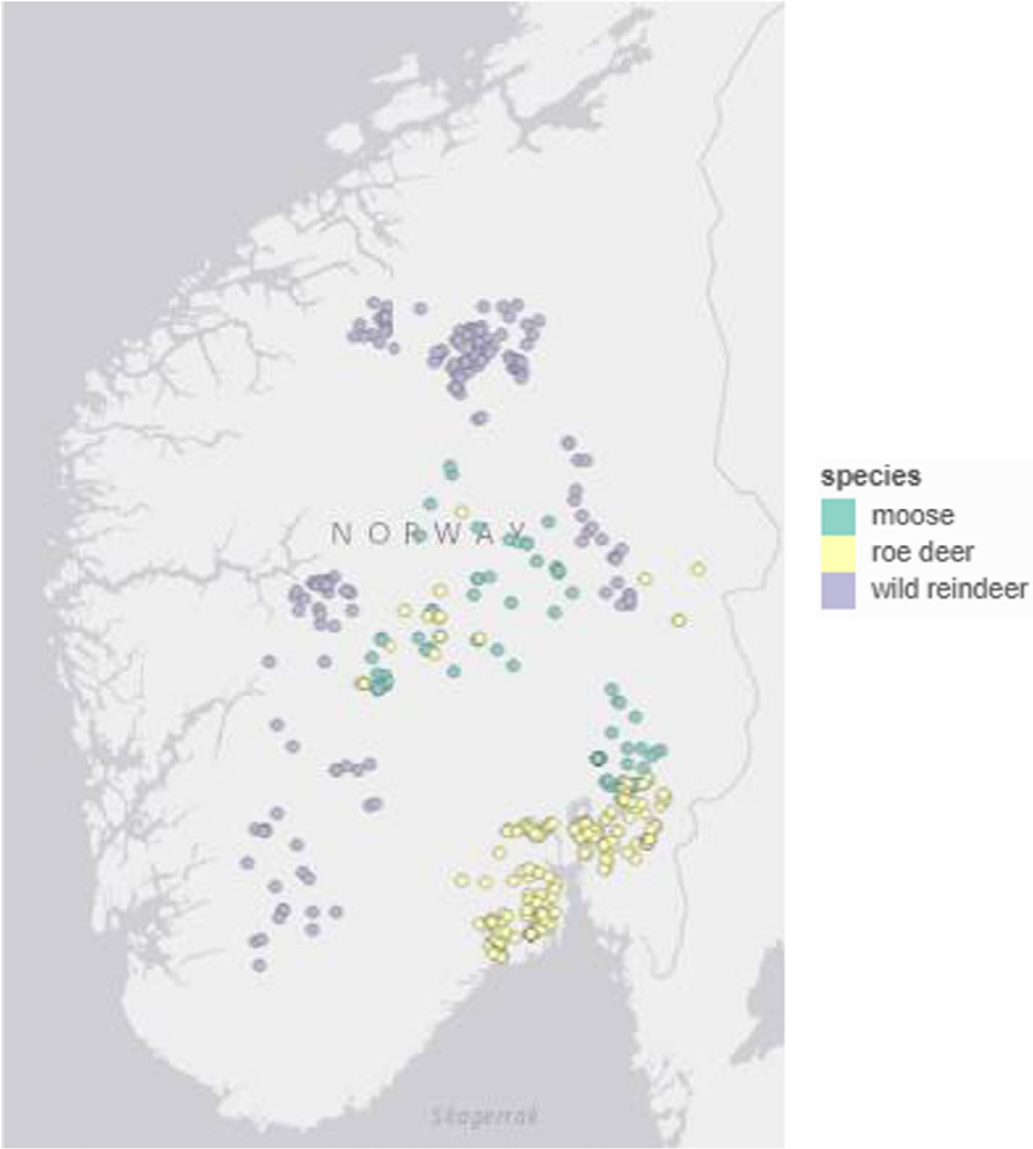
Map displaying the distribution of the opportunistic data for each species

Because of the low number of opportunistic records directly within the telemetry study sites of both roe deer and moose, we built a 10 km buffer around the distribution of telemetry observations and included records inside the buffer surrounding the telemetry data and inside the distribution of the telemetry data. For wild reindeer, enough opportunistic records were available within the areas from which telemetry locations were available (in the mountainous areas in the south, ranging from the southern part of Trøndelag and a county southward) and no buffer was needed. Because our paper aims to provide a general method, we aimed to get a dataset as representative as possible of the “general” or “everyday” citizen scientist. We sorted all opportunistic observations by the name of the observer and deleted observations made by “super‐observers,” persons contributing to more than 50% of the dataset. These super‐observers were often employed by wildlife management institutions, and therefore, their observations were not considered to be representative of typical opportunistic data. The resulting dataset was composed of 160 opportunistic records for moose, 316 for roe deer and 183 for wild reindeer. The spatial distribution of the observations used in our analysis is displayed in Map 1.

#### 2.1.3 Explanatory variables

We first fitted an observer model to quantify the biases in opportunistic data using variables related to human infrastructure (that might influence observer access to wildlife habitat), human activities (that might influence the potential number of observers), and habitat associated visibility (that might influence the detectability of a species to an observer), which are factors that are presumed to be the main drivers of biases in opportunistic records (Geldmann et al., 2016; Tiago et al., 2017).

After sampling availability locations with regard to the observer model, we estimated habitat selection for moose, roe deer, and wild reindeer using explanatory variables related to habitat (i.e., environmental data) and to human activity.

##### Explanatory variables for the observer model

We extracted map layers on roads and human settlement from Open Street Map (https://www.openstreetmap.org; OSM) and Statistics Norway (https://kart.ssb.no/), respectively. Human settlements are defined as a cluster of buildings inhabited by at least 200 persons and the distance between buildings is less than 50 m (https://www.ssb.no/en/klass/klassifikasjoner/110). In the OSM dataset, we selected the main segments of the road network: motorway, trunk, primary, secondary, tertiary, unclassified, and residential. Datasets were then used to compute 10 m resolution rasters of distance to roads and distance to human settlements. Both rasters were created in ArcGIS Pro.

To represent human activity intensity, we used path use intensity and population number. Path use intensity captures the number of human activities such as running, cycling, or hiking events occurring on a given path. We aggregated all variables to a resolution of 1 km × 1 km, that is, the finest common resolution across covariates. For both moose and roe deer, we used Strava Metro data (https://metro.strava.com/) for southern Norway to compute a path use intensity raster. The Strava Metro product is a shapefile composed of OSM trails and roads. In its attribute table, each segment contained the number of users who recorded an activity, and calibration of the STRAVA activity counts using fixed‐point counter station estimates revealed a strong overall correlation (Venter et al., 2020). Data were available between 2017 and 2020. We summed the number of users who recorded an activity within 1 km × 1 km grid cells and rasterized the results. Because of a lack of Strava users in high mountain habitats (due to poor telephone network coverage and battery constraints on mobile devices), we used a trail use index derived from trail counter data (automatic devices that record the number of people passing) (Gundersen et al., 2019). Human population density (residential) at a resolution of 100 m × 100 m was extracted from the national database and summed within 1 km × 1 km grid cells.

Finally, the Corine Land Cover dataset (available at a 100 m × 100 m resolution raster) was used to calculate the amount of forested area within a 1 km × 1 km grid cell as a proxy for visibility. We assumed that the more forested area in a grid cell, the harder it would be for an observer to spot an animal.

##### Explanatory variables for the resource selection function

The explanatory variables used to estimate the resource selection function were chosen based on previous fine‐scale studies of habitat selection of these species (for roe deer, see Bouyer et al., 2015; for moose, see Bjørneraas et al., 2011, 2012; and for wild reindeer, see Panzacchi et al., 2015; Table 1). Slope and altitude were computed from a 20 m resolution Digital Elevation Model extracted from the Norwegian Spatial Data Infrastructure (https://www.geonorge.no/). Path use intensity, distance to roads, and urban settlements and forest coverage were the same variables we used in the observer model. We also included agricultural area coverage, which was computed by filtering the pixels labeled “agricultural areas” in the Corine Land Cover dataset. We then calculated the proportion of agricultural area in each 1 km × 1 km grid cell.

**TABLE 1:** Description of the covariates used in the observer model and in the estimation of the resource selection function

Variables	Explanation	Resolution	Observer model	RSF
*Environment*				
alt	Mean altitude in each pixel	50 m × 50 m	No	Yes
slope	Mean slope in each pixel	50 m × 50 m	No	Yes
n_forest	Proportion of forested areas within each pixel	1 km × 1 km	Yes	Yes
*Human activity*				
d_roads	Distance to roads	10 m × 10 m	Yes	Yes
d_urb	Distance to human settlements	10 m × 10 m	Yes	Yes
path_use	Number of users who recorded an activity in each pixel	1 km × 1 km	Yes	Yes
n_agr	Proportion of agricultural fields within each pixel	1 km × 1 km	No	Yes
pop	Number of inhabitants in each pixel	100 m × 100 m	Yes (no reindeer)	No

#### 2.1.4 Sampling availability locations

##### Sampling random availability locations

Resource selection functions are commonly used to characterize species’ habitat use (Boyce & McDonald, 1999). RSFs are used to compare environmental covariates at locations visited by an animal with environmental covariates at a set of locations assumed to be available to the animal (Manly et al., 2007). Concretely, RSFs are presence/background (or presence/pseudo‐absence) species distribution models used at a higher order of selection (Johnson, 1980) and are evaluated by fitting a logistic regression to observed and available locations with available locations consisting of points sampled randomly or systematically from within an animal's estimated home range (Manly et al., 2007).

Sampling available locations is a crucial step in habitat selection studies, and different choices of available locations may influence the quantification of selection (Beyer et al., 2010). Usually, areas are defined as “available” if they are found within a minimum convex polygon (MCP) drawn around the area from which “use” locations are derived (Calenge, 2011). We consequently randomly sampled available points from a uniform distribution for opportunistic and telemetry observations from within the MCP built around the telemetry observations.

#### 2.1.5 Sampling availability locations with regards to observation biases

Although areas are theoretically available, they are not all equally accessible and observable to the citizen scientist within any given part of the species’ range. Drawing availability locations at random implies the assumption of homogeneous accessibility throughout the species’ home range. This assumption is, however, not realistic as citizen scientists’ movements are influenced by a variety of factors. Not accounting for factors influencing the probability of citizen scientists being in a specific area could lead to biases in the parameters estimated by any analysis such as an RSF (Sicacha‐Parada et al., 2020).

We represented the spatial biases contained in opportunistic observations due to the observer behavior using a model of the biases known here as the *observer model* (Table 2). The observer model estimates which factors influence the probability of an opportunistic observation being in a specific location. We use telemetry observations as a baseline and compare the differences in different locations of the telemetry data and opportunistic observations. For both telemetry and opportunistic data, we extracted at each location and for each observation the value of the covariate that has been demonstrated to influence the observation process (Table 1). We then fitted a logistic regression, the response variable being record type (i.e., the probability that an observation with certain environmental characteristics was opportunistically collected rather than derived from telemetry; opportunistic records were coded as 1 and telemetry observations coded as 0) and the explanatory variables being the extracted covariate values. If estimated parameter values (*β*) in Equation (1) are different from 0, then there is mismatch in the environmental space for the variables we are testing between opportunistic observation locations and telemetry locations. For instance, if the mean distances to roads are lower for the locations of opportunistic observations than the telemetry observations, the observer model would return a negative parameter value.

(1) $$\begin{array}{cc} \text{Record type} & =\alpha +\beta_{1}\;\text{distance to roads}+\beta_{2}\;\text{distance to urban centers}\\ & \;+\beta_{3}\;\text{path use intensity}+\beta_{4}\;\text{forest coverage}+\beta_{5}\;\text{population} \end{array}$$

**TABLE 2:** Terms specific to the method presented in this study and their associated definitions

Term	Definition
Observer model	Model quantifying accessibility within species home range for a citizen scientist by evaluating differences in locations between opportunistic and telemetry data.
Corrected availability	Available locations sampled and that are used in the corrected OPP model in tandem with opportunistic observations.
Corrected OPP model	Resource selection function estimated by an infinitively weighted logistic regression using both corrected availability locations and opportunistic observations.
Naïve OPP model	Resource selection function estimated by an infinitively weighted logistic regression using both availability locations randomly sampled across the species’ home range and opportunistic observations.

We then randomly sampled 100,000 locations within the polygon surrounding the telemetry points (plus buffer) and predicted the probability that a point would be “used” by the citizen scientist based on the parameter values estimated by the “observer model.” Finally, we sampled the corrected availability locations (n = 3 x opportunistic citizen science observations, Table 2, Muff et al., 2020) from the opportunistic citizen science observations (OPP) “use distribution” to estimate the RSF.

### 2.2 Simulation study

#### 2.2.1 Simulating environmental variables

We created a simulated landscape by generating different environmental variables with the nlmr package in R (Sciaini et al., 2018) on a grid composed of 200 × 200 regularly spaced cells. *Distance to human settlements* was generated by calculating the distance from each grid cell to a location placed on the upper left corner of the study area. *Forest* and *nice viewpoints* were simulated as clusters across the simulated landscape and each grid cell was coded as 0 for “absence of forest” or “absence of nice viewpoints” and 1 as “presence of forest” or “presence of nice viewpoints” (Saura & Martínez‐Millán, 2000). For *Other gradient (*a hypothetical variable which could represent another road or some properties of the study area such as a gradient of vegetation*)* and *distance to roads*, we generated a segment across the landscape and the distance to that segment was computed for each grid cell. It should be noted that the variable *Other gradient* was generated to be highly correlated with *distance to roads* (Pearson's *r* = −.80). While distance to roads is directly associated with both the probability of occurrence of a species and an observer being present in a specific grid cell, other gradient is only associated with the probability of occurrence of the species being present in a specific grid cell. By being correlated to distance to roads, we could test whether sampling locations with regard to the observer model (i.e., including distance to roads) could correct for a variable not included in the observer model (i.e., other gradients). Finally, we simulated *elevation* across the landscape using a Gaussian random field (mean = 1 and std = 1).

#### 2.2.2 Simulating animal locations and opportunistic observations

We simulated animal locations which were linearly dependent on the values of the simulated environmental conditions using Equation (2). More specifically, using the following parameterization, we simulated a species that was more likely to be situated in forests (*β*_forests_ = 2.5), in lower altitude (*β*_altitude_ = −2), away from roads (*β*_d_roads_ = 4.5) and attracted by an unknown gradient (*β*_other_gradient_ = 4.5). The probability of presence of the species in a specific grid cell is given by Equation (2).

(2) $$\text{logit}\left(\pi \right)=‐7+\beta_{\text{forest}}*\text{forest}+\beta_{\text{altitude}}*\text{altitude}+\beta_{d_{\text{roads}}}*d_{\text{roads}}+\beta_{{\text{other}}_{\text{gradient}}}*{\text{other}}_{\text{gradient}}$$

The likelihood of an observer being present in a given area depends on multiple variables such as accessibility (Sicacha‐Parada et al., 2020). We gave a probability score to each grid cell, the higher the score, the more likely an observer is to be present. A high score (i.e., high probability of an observer being present) was given to grid cells located close to roads (*β*_d_roads_ = −6), close to densely populated areas (*β*_d_urb_ = −3) and if there were nice viewpoints (*β*_nice_viewpoints_ = 1). The calculation of the probability of presence score (*α*) is given by Equation (3).

(3) $$\text{logit}\left(\alpha \right)=\beta_{d_{\text{roads}}}*d\_\text{roads}+\beta_{d_{\text{urb}}}*d\_\text{urb}+\beta_{{\text{nice}}_{\text{viewpoints}}}*\text{nice}\_\text{viewpoints}$$

Finally, for each grid cell we calculated the probability ψ of having an opportunistic observation. We defined ψ as the product of *α*, the probability of an observer being within the area represented by the grid cell and π, the probability of presence of the species being within the area represented by the grid cell. Thus, the probability of having an opportunistic observation in a specific grid cell was higher if the grid cell was easily accessible and if the probability of presence of the species was high (Equation 4).

(4) ψ=απ

### 2.3 Analysis

#### 2.3.1 Model fitting

For each species, we estimated an RSF with (1) telemetry data and randomly sampled availability points within the MCP drawn around the telemetry locations, (2) opportunistic citizen science data and availability points randomly sampled within the MCP surrounding all observed locations, and (3) opportunistic citizen science data with availability points sampled with regard to the “observer model” (see Section 2.3.2). In the following, (1) is referred to as the *telemetry model*, (2) as the *RSF OPP naïve model*, and (3) as the *RSF OPP*‐*corrected model* (Table 2). To account for individual‐specific variation in the telemetry dataset, we used a random slope for all coefficients (Muff et al., 2020).

Following results and recommendations from previous studies (Muff et al., 2020), we fit a logistic regression in which the background points were assigned a weight of 100. Theoretically, results from an infinitively weighted logistic regression are similar to those of a logistic regression with a large number of availability points (i.e., at least 10 times the number of presence locations, Muff et al., 2020). Using an infinitively weighted logistic regression thus reduces computational burden and is less subject to under‐sampling of availability points (Fithian & Hastie, 2013; Muff et al., 2020).

We used the integrated nested Laplace approximation (INLA) approach with the package R‐INLA for all models (Lindgren & Rue, 2015). INLA is a faster alternative to Markov chain Monte Carlo approaches and yields similar, if not identical, results (Beguin et al., 2012). We log‐transformed all variables except for slope because of their skewed distributions. All statistical analyses were conducted in R version 4.0.2 (R Core Team, 2020).

### 2.4 Habitat suitability maps

In order to visualize the consequences of different background selection approaches, we predicted habitat suitability maps on a grid placed over the MCP drawn around the telemetry locations of each species. These maps were produced using the covariates described earlier and the mean parameters of the RSFs for each of the three approaches.

## 3 RESULTS

### 3.1 Difference in the locations of opportunistic citizen science observations and animal telemetry

**FIGURE 2:**
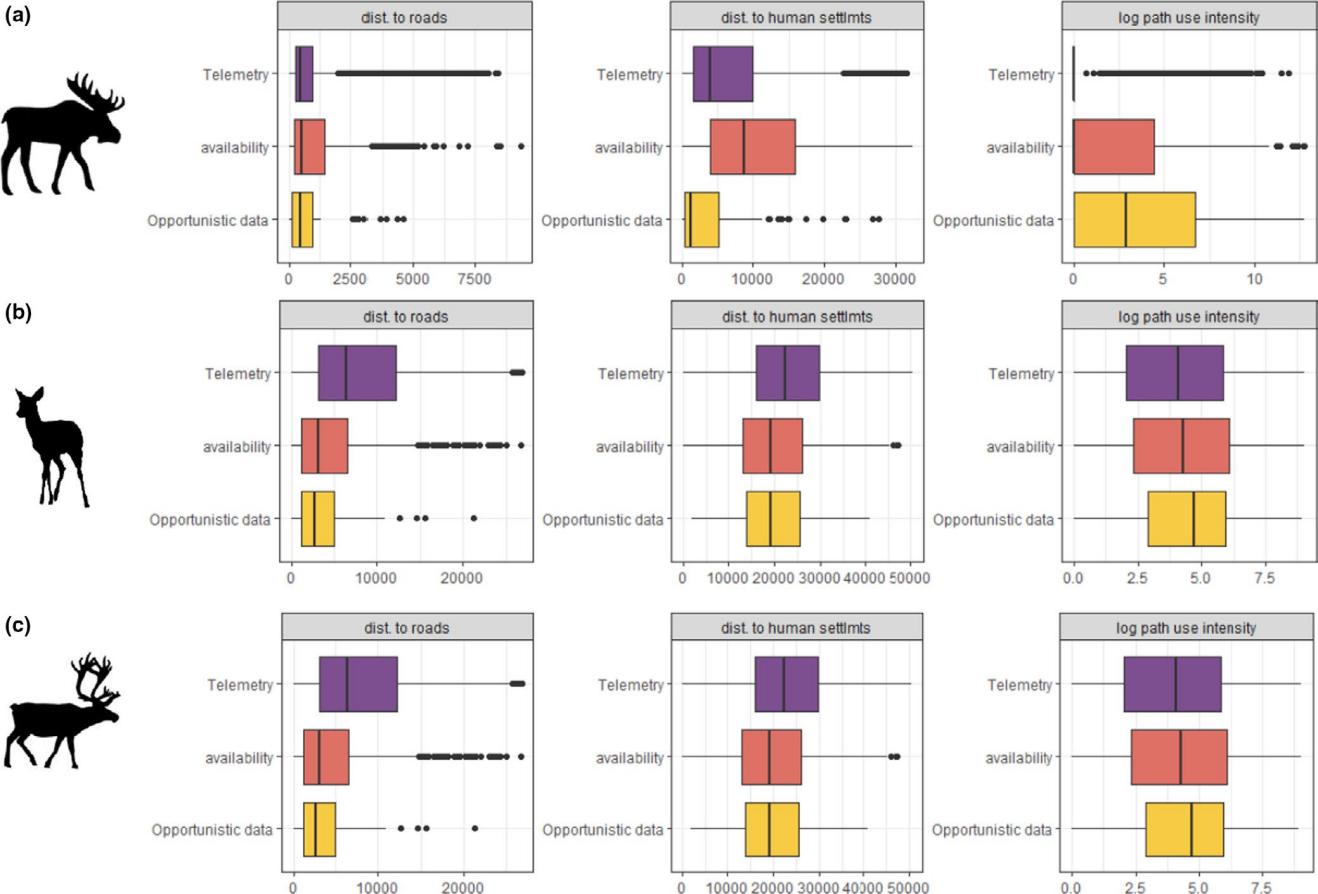
Boxplots of the distribution of telemetry locations, availability locations, and opportunistic citizen science observations within the distance to roads, distance to human settlements, and path use intensity spectrum for (a) moose, (b) roe deer, and (c) wild reindeer

We first estimated the potential biases in opportunistic observations by comparing them with telemetry data and available locations within the species’ home range. Figure 2 shows that human activity variables (i.e., distance to roads, distance to human settlements, and path use intensity) influence the location of opportunistic observations and telemetry observations differently and that the contrast between telemetry and opportunistic data is species’ specific. While opportunistic and telemetry locations are similarly distributed with regard to distance to roads for moose (mean_dist to roads moose_ =741 and 757 m for opportunistic and telemetry locations, respectively), summary statistics indicate that opportunistic observations are on average closer to the roads for both roe deer and wild reindeer (mean_dist to road roe deer_ = 262 and 422 m and mean_dist to roads wild reindeer_ = 3574 and 8283 m for opportunistic and telemetry locations, respectively).

In contrast, opportunistic observations are on average closer to human settlements than telemetry observations for all species, although the discrepancy is particularly strong for moose (mean_dist to human settlements moose_ = 3693, 6842; mean_dist to human settlements roe deer_ = 2222, 4022; mean_dist to human settlements wild reindeer_ = 19,417, 23,536 for opportunistic and telemetry locations respectively). Finally, the descriptive statistics show that opportunistic observations are located on average closer to more utilized paths than telemetry locations for all three species (mean_log path use intensity moose_ = 3.54, 1.06; mean_log path use intensity roe deer_ = 7.43, 6.77; mean_log path use intensity wild reindeer_ = 4.41, 3.91 for opportunistic and telemetry locations respectively).

### 3.2 Results of the simulation study

The simulation study was used to test our method under ideal conditions and confirm the intuition that accounting for biases reduces error in inference.

**FIGURE 3:**
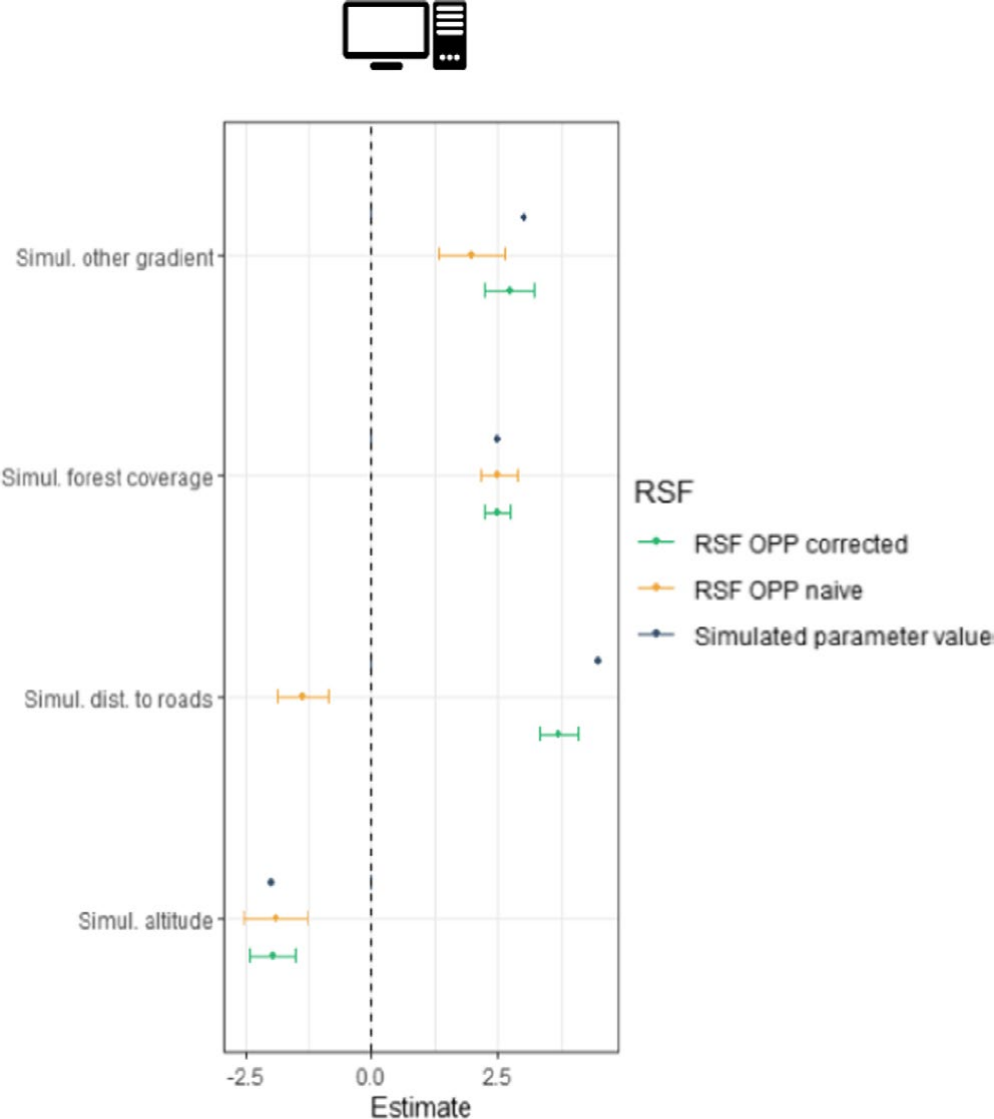
RSF parameter estimates obtained in the simulation for the naïve OPP model (in yellow) and the corrected OPP model (in green). Blue dots represent the parameter value used to simulate species presence across the simulated landscape. Dots represent the mean parameter estimates and bars the 95% credible intervals

The results of the simulation (displayed on Figure 3) show that the model accounting for observer bias by sampling corrected available locations (i.e., RSF OPP corrected) returns coefficients that are more consistent with the parameter values used to simulate the species presence (i.e., simulated parameter value in Figure 3) than the model sampling random availability locations (i.e., RSF OPP naïve). This is particularly visible for the simulated distance to roads where the RSF CS naïve model returns a parameter value of opposite direction (*β*_Simul. dist. to roads RSF OPP naive_ = −2.098). In contrast, the *β*_Simul. dist. to roads RSF OPP corrected_ = 3.699, which is consistent with and close to the simulated parameter value of 4.5.

The parameter estimate for other gradients seems to benefit from the correction in availability as the mean estimated parameter value is close to the true parameter value of 3 used to simulate species presence (*β*_Simul other gradient RSF OPP naïve_ = 1.438, *β*_Simul other gradient RSF OPP corrected_ = 2.535).

Finally, our simulation suggests that both the corrected RSF and naïve RSF return estimated parameter values that are very similar to the parameter value used to simulate species presence for variables influencing species occurrence only (i.e., *β*_forest_ = 2.5, 2.471, 2.328 and *β*_altitude_ = −2, −1.617, −1.496 for the parameter value used to simulate species’ presence, RSF OPP naïve, and RSF OPP corrected, respectively).

### 3.3 Results from modeling the biases in opportunistic observations: the observer models

Even though the extent of the biases was qualitatively examined in Section 3.1, the method we suggest in this paper relies on the quantification of these biases. We quantified the biases by specifying an observer model and estimating the strength of the variables influencing the accessibility within the species’ home range for a citizen scientist.

**FIGURE 4:**
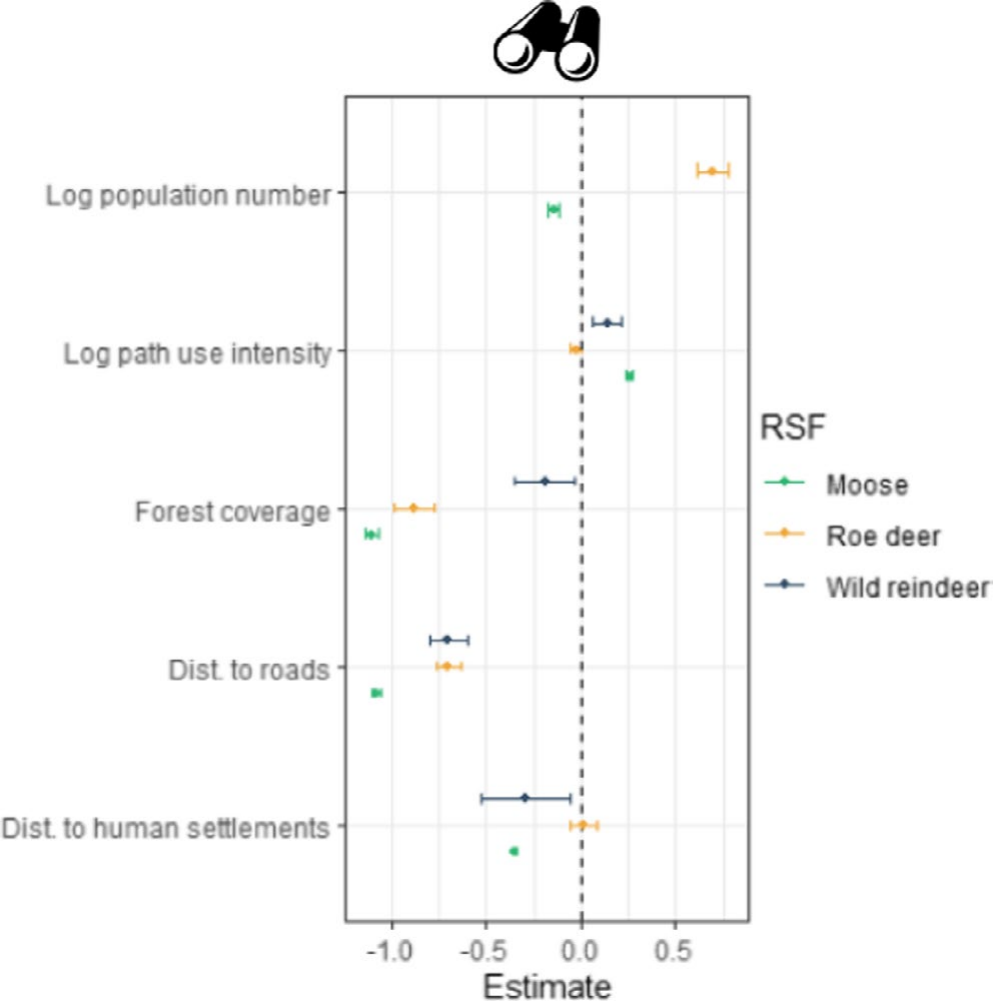
Parameter estimates from the moose, roe deer, and wild reindeer observer models. The further away the estimate is from 0, the more OPP observations are affected by the variable compared to telemetry observations. Dots represent the mean parameter estimate and bars the 95% credible intervals

The observer model (Figure 4) confirms that there are spatial discrepancies between opportunistic citizen science data and telemetry locations. Results indicate that opportunistic observations are on average located within a different environmental space. Nevertheless, while there are similarities between species, the extent to which the different variables influence opportunistic observation compared to telemetry observations differs. Opportunistic observations are on average closer to the roads than telemetry locations for both roe deer and wild reindeer (mean_dist. to roads_ = −0.700, −0.729 for roe deer and wild reindeer respectively), but we only found a very weak effect for moose (mean_dist to roads_ = −0.027). Nevertheless, opportunistic observations are closer to human settlements than telemetry locations for moose (mean_dist to human settlements_ = −0.405).

The observer models show that opportunistic observations are more likely to be located near a heavily utilized path than telemetry locations for moose and wild reindeer mean_log path use intensity_ = 0.247, 0.147 for moose and wild reindeer, respectively). It does not make a difference for roe deer, where opportunistic observations were located with a similar frequency as telemetry locations regarding path use intensity (mean_log path use intensity_ = −0.027). Opportunistic observations of moose and roe deer are also likely to be made in areas with higher human density than telemetry locations (mean_log population number_ = 0.204, 0.700).

Finally, visibility (i.e., approximated by forest coverage within a grid cell) also had a major role as opportunistic observations for all three species were more likely to be found in grid cells containing less forests (mean_Forest coverage_ = −0.771, −0.876, −0.149 for moose, roe deer, and wild reindeer, respectively).

### 3.4 Resource selection function obtained with a model using telemetry data, opportunistic data with random availability, and opportunistic data using a corrected availability

Finally, after quantifying the extent of the biases in 3.3 and sample availability locations regarding the observer model, we were able to estimate and compare the RSFs for the different models.

**FIGURE 5:**
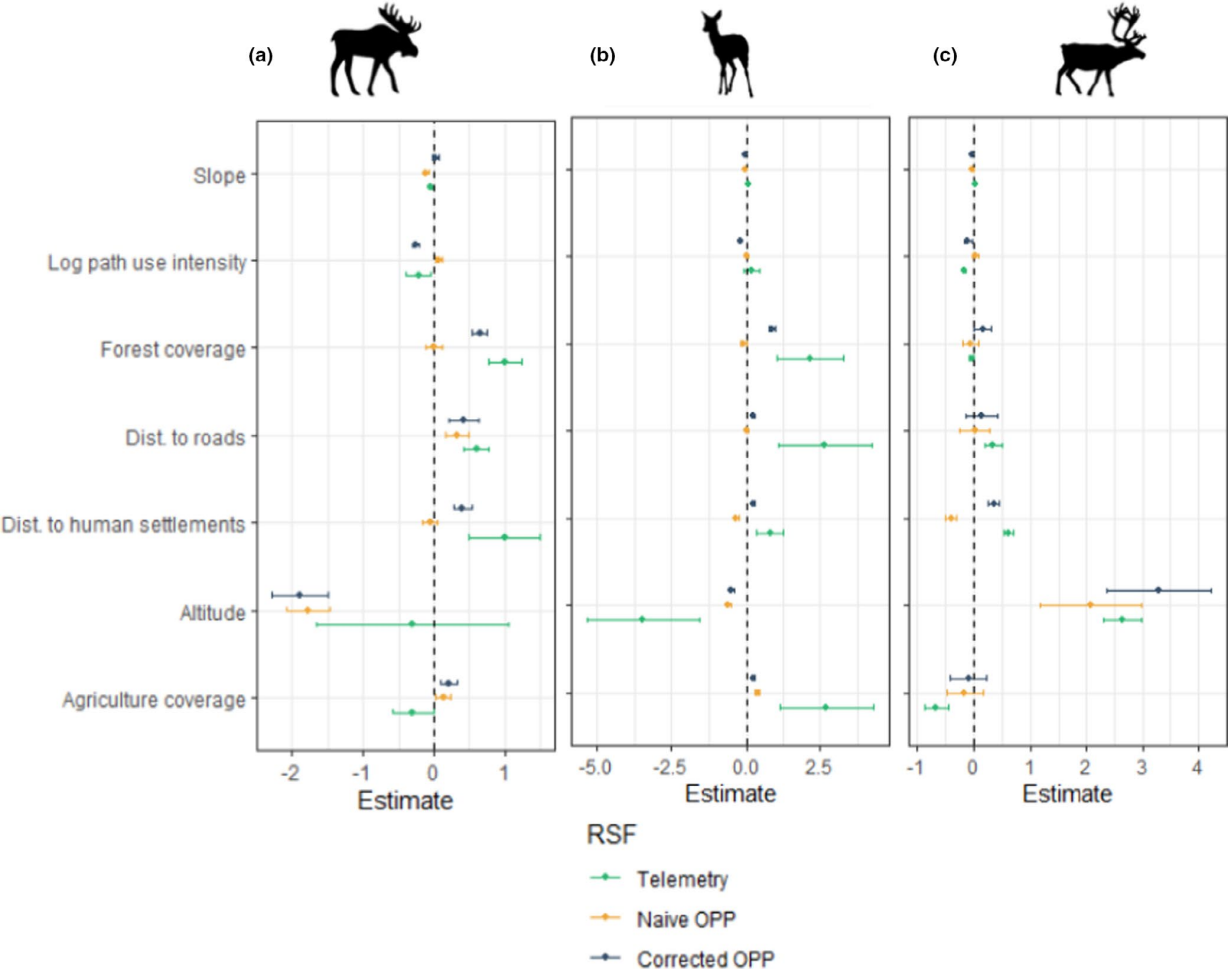
RSF parameter estimates for (a) moose, (b) roe deer, and (c) wild reindeer. In green are the parameter estimates obtained with the telemetry model, in yellow with the naïve OPP model, and in purple with the corrected OPP model. Dots represent the mean parameter estimates and bars the 95% credible intervals

Discrepancies between the opportunistic citizen science observations and the telemetry locations had substantial effects on the naive OPP model (Figure 5). Especially, in the model using telemetry data the coefficient was positive for distance to roads and distance to human settlements for all three species (mean_dist to roads telemetry_ = 0.604, 0.779, 0.613, mean_dist. human settlements telemetry_ = 1.005, 2.653, 0.341 for moose, roe deer, and wild reindeer, respectively). In contrast, the coefficients for distance to roads were negative for roe deer and wild reindeer (mean_dist. to roads naïve CS_ = −0.036, −0.406 for roe deer and wild reindeer, respectively) in the naïve OPP model and negative for distance to human settlements for moose (mean_dist. to human settlements naïve CS_ = −0.055). In the telemetry model, we can also see that the coefficient for path use intensity is negative for both moose and wild reindeer (mean_log path use intensity telemetry_ = −0.219; −0.166 for moose and wild reindeer, respectively), but in the naïve OPP model this value is positive for moose and very close to 0 for wild reindeer (mean_log path use intensity telemetry_ = 0.066 and 0.029 for moose and wild reindeer, respectively). Finally, the coefficient for the proportion of forested area per grid cell is positive in the telemetry model for moose and roe deer (mean_forest coverage telemetry_ = 1.001; 2.155 for moose and roe deer, respectively) while it is close to 0 in the naïve OPP model for both species (mean_forest coverage naïve CS_ = 0.004; −0.099 for moose and roe deer, respectively).

Interestingly, correcting for the availability points used in the RSF brings the coefficients closer to the telemetry coefficients in most cases. Particularly, in the corrected OPP model the sign of the coefficients for distance to roads and distance to urban settlements is consistently in the direction of the telemetry ones for all three species (mean_dist. to roads corrected CS_ = 0.419, 0.240, 0.359, mean_dist to human settlements corrected CS_ = 0.409, 0.216, 0.137 for moose, roe deer, and wild reindeer, respectively). The coefficient for path use intensity is also of the same sign of the telemetry model for moose and wild reindeer (mean_log path use intensity corrected CS_ = −0.248, −0.103 for moose and wild reindeer, respectively) while the coefficient for forest cover has the same sign as the telemetry value for moose and roe deer.

While the corrected model is more consistent with the telemetry model than the naïve model for most of the coefficients, surprisingly we see in Figure 5 that the corrected OPP model coefficients do not get closer to the telemetry model for either proportion of agricultural coverage or altitude and even seems to perform worse than the naïve OPP model for these variables (mean_agricultural coverage corrected CS_ = 0.204, 0.217, −0.099, mean_altitude corrected CS_ = −1.896, −0.493, 3.297 for moose, roe deer, and wild reindeer, respectively).

### 3.5 Suitability maps obtained with a model using telemetry, opportunistic with random availability, and opportunistic using a corrected availability

**FIGURE 6:**
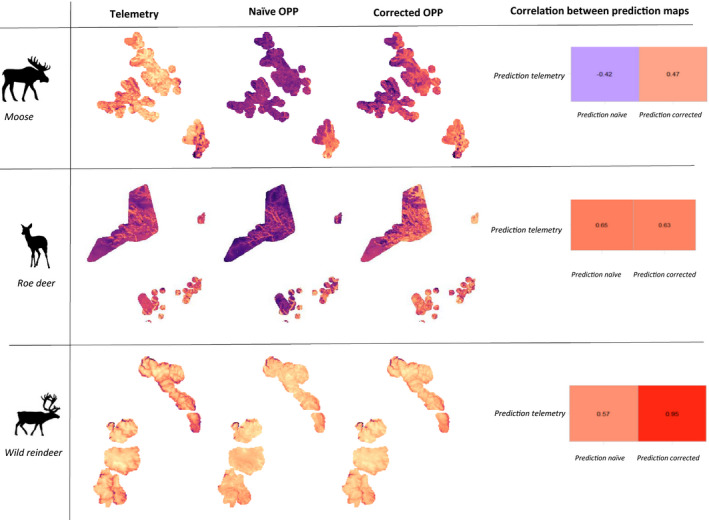
Suitability maps obtained using the mean coefficients of the telemetry (column 1), naïve OPP (column 2), and corrected OPP models (column 3) for roe deer (row 1), moose (row 2) and wild reindeer (row 3). Map values are the log‐odds probabilities. On row 4 are the Pearson correlation coefficients between the habitat suitability maps obtained with the telemetry model and the ones obtained with the naïve OPP model and the corrected OPP model

The habitat suitability maps obtained with the naïve OPP model are similar to the habitat suitability maps obtained with telemetry location for both roe deer and wild reindeer (Figure 6). In fact, the Pearson correlation coefficients between the habitat suitability maps are 0.65 and 0.57, respectively, which can be considered as a moderately strong positive correlation. However, for moose the naïve OPP model does not represent well the habitat suitability map obtained with telemetry locations as the correlation coefficient is negative (*r* = −.42).

The corrected OPP models result in a suitability map more correlated with the predictions from the telemetry model than the naïve OPP model for both moose and wild reindeer and as good as the naïve OPP model for roe deer. The improvement is particularly obvious for the moose, for which the corrected OPP model return a moderately strong positive correlation with the habitat suitability map obtained with telemetry locations (*r* = .47). Furthermore, for wild reindeer the corrected OPP model returns a habitat suitability map strongly correlated to a suitability map obtained with telemetry locations (*r* = .95).

## 4 DISCUSSION

In this study, we show that it is possible to infer habitat selection of a species in an area rich in opportunistic data but where relatively little telemetry data is available and that opportunistic citizen science observations are skewed toward areas that are more easily accessible and used by humans (i.e., the citizen scientists). Our results show that opportunistic observations are on average closer to human infrastructure and highly frequented trails than telemetry observations. This is are consistent with many studies pointing out the biases in opportunistic data (Geldmann et al., 2016; Sicacha‐Parada et al., 2020; Tiago et al., 2017). Because of these biases, a naive use of opportunistic observations in fine‐scale habitat selection studies can lead to misleading results. We show how the RSF obtained with a naïve model based on this type of data can return parameter values inconsistent with the species habitat preferences as estimated from telemetry data. However, implementing an observer model that accounts for the degree of accessibility to potential citizen observers within the species’ home range provides capacity to account for these biases.

Under ideal conditions (i.e., the simulation), our method returns parameter estimates consistent with the values used to simulate species’ presence data. Empirically, an RSF estimated with our correction method returns parameter values consistent with an RSF based on telemetry. While the correction seems less helpful for roe deer, it gave substantial improvement for species that are more sensitive to human activities such as moose and wild reindeer. In fact, except for a few parameters, the estimates had consistently the same direction and were within the 95% credible interval of the telemetry models’ parameter estimates. Drawing habitat suitability maps from the RSF OPP‐corrected model also drastically improved the consistency with the suitability maps drawn from the telemetry data, with the exception for roe deer, which was already quite good. In contrast, the RSF OPP naïve model returned habitat maps that, in the worst cases, were totally misleading (e.g., moose in our study). Even though the method clearly shows that there is potential for using corrected opportunistic data for fine‐scale habitat selection studies, we can see some concerns notably with the estimated parameter values for both altitude and agricultural field coverage which do not get closer to the coefficients of the telemetry model. In the simulation, the RSF OPP‐corrected model should always correct coefficients related to any bias variables and the discrepancies might only be due to the stochasticity inherent to any statistical model, so that bootstrapping could potentially be used to stabilize the estimates (see Figures S1 and S2). Alternatively, these results may be due to our failure to correctly understand how these environmental layers influence citizen scientists’ movements and observation pattern.

Finally, the suggested method relies on a good estimation of observer bias. This requires reliable information about the species’ ecology which can be obtained with GPS‐telemetry data. Nevertheless, GPS‐telemetry studies are costly and thus cannot be conducted everywhere on all species. Preliminary work (described in the annexes) suggests that using telemetry observation from an auxiliary species with similar habitat preference to a target species could be used to estimate observer bias, correct for availability locations used in the logistic model, and thus partially correct parameter estimates (see Figures S6–S11).

There are multiple reasons for why opportunistic observations do not accurately produce resource selection functions and more generally reflect species’ ecology including spatial and temporal biases (Isaac et al., 2014). While the method suggested in this paper account for these biases to improve ecological inference, it is also necessary to improve opportunistic data collection. Citizen scientists usually report observations from quite human‐dominated areas, or for instance when surprised to find a species in a place where they are not used to be seen, capturing only certain species’ individual behavior that are not representative of the species usual range. Encouraging other types of citizen scientists, such as hunters or other outdoor enthusiast, could improve the coverage of the dataset and improve inference (Cretois et al., 2020).

Despite its limitations, our method is a first step toward improving the use of opportunistic data in habitat selection studies. In fact, we do not present our method to correct for availability as an infallible technique but rather as a way to initiate conversations and research among ecologists to account for spatial biases in opportunistic data for more accurate inference at fine scales. Methods to account for variation in the observation process in opportunistic observations are developing and improving, notably with the potential of occupancy models (Altwegg & Nichols, 2019; Strien et al., 2013) and integrated models (Isaac et al., 2020). Nevertheless, these developments account for biases in opportunistic data at the distribution level (4th order of selection; Johnson, 1980), and to our knowledge, our study is the first attempting to find a general solution for using opportunistic data at finer scale. Instead of using telemetry data to infer biases in opportunistic data, it would also be possible to use other independent and reliable data sources such as observations systematically collected by professionals. It should be noted that our work is preliminary, and could be easily expanded. For instance, a possible extension of our method would involve using auxiliary species observation models to correct the habitat preference model of a target species (Figures S11–S13).

## 5 CONCLUSION

In this paper, we explore the challenges and the opportunities of using opportunistically collected citizen science data in habitat preference studies. We show that opportunistic data used in a naïve way can be misleading and result in spurious ecological inference. Accounting for the observation process reduces this risk. Our study is a first step toward using opportunistic data for finer scale habitat analyses.

## CONFLICT OF INTEREST

The authors declare no conflict of interest.

## AUTHOR CONTRIBUTIONS

**Benjamin Cretois:** Conceptualization (lead); Data curation (lead); Formal analysis (lead); Methodology (lead); Visualization (lead); Writing‐original draft (lead). **Bram van Moorter:** Conceptualization (lead); Formal analysis (supporting); Writing‐original draft (supporting). **John D. C. Linnell:** Conceptualization (lead); Writing‐original draft (supporting). **Emily G. Simmonds:** Formal analysis (lead); Writing‐original draft (supporting). **Christer M. Rolandsen:** Writing‐original draft (supporting). **Erling J. Solberg:** Writing‐original draft (supporting). **Olav Strand:** Writing‐original draft (supporting). **Vegard Gundersen:** Writing‐original draft (supporting). **Ole Roer:** Writing‐original draft (supporting). **Jan Ketil Rød:** Writing‐original draft (supporting).

### OPEN RESEARCH BADGES

This article has been awarded Open Data, Open Materials Badges. All materials and data are publicly accessible via the Open Science Framework at https://zenodo.org/record/4590153#.YGGdMq8zZaQ and https://github.com/BenCretois/CS_in_habitat_studies.

## Supporting information

Figures S1‐S13

## Data Availability

Code and data to run the analysis and the simulation study can be found on Zenodo, https://doi.org/10.5281/zenodo.5520616. A more in‐depth description of the code can be found on https://github.com/BenCretois/Cretois_EE_2021.
