# Supplementary material for: Identifying and correcting spatial bias in opportunistic citizen science data for wild ungulates in Norway
Source: Ecol Evol. 2021 Oct 5;11(21):15191–204. doi: 10.1002/ece3.8200 (PMC8571602; doi:10.1002/ece3.8200)
Supplement: Supplementary file 1 — Figures S1‐S13 [file ECE3-11-15191-s001.docx]

Annexes for:

Identifying and correcting spatial bias in citizen science data for wild ungulates in Norway

This document includes:

Supplementary material and method

Figure S1: Maps of the simulated citizen science observations and species locations.

Figure S2: Maps of the simulated landscape.

Figure S3: Proportion of times that the covariate estimate was improved by the corrected model. Results from 100 bootstraps of the data generation process.

Figure S4. Proportion of times that the covariate estimate was improved by the corrected model. Results from 100 bootstraps of the background point selection process.

Figure S5. Histograms describing the distribution of the covariates used in the moose observer model and the moose RSF

Figure S6. Histograms describing the distribution of the covariates used in the roe deer observer model and the roe deer RSF

Figure S7. Histograms describing the distribution of the covariates used in the wild reindeer observer model and the wild reindeer RSF

Figure S8. RSF parameter estimates for moose

Figure S9. RSF parameter estimates for roe deer

Figure S10. RSF parameter estimates for wild reindeer

Figure S11. Moose suitability maps

Figure S12. Roe deer suitability maps

Figure S13. Wild reindeer suitability maps

**Supplementary material and method**

*Simulation set up*

See main manuscript section 2.2

*Simulation to demonstrate effectiveness of the model framework*

*Quantification of simulation variability*

The data generation process of the simulation is stochastic. To represent the uncertainty and variability introduced from this simulation process, we reran the data generation process to create 100 different realisations of the simulated environmental and occurrence data. We fit all of the models (telemetry, naïve and corrected RSF) to each simulated dataset and recorded if the estimates of the coefficients in the final models were improved by the correction. Improvement of the coefficient estimates is the primary aim of our proposed modelling framework and therefore we are most concerned with uncertainty in this element. Improvement was indicated as true or false, where true arises when the coefficient estimate from the corrected model was closer to the telemetry model coefficient estimate than the naïve model estimate. No measure of strength of improvement was included.

The results show that improvements in the coefficient estimates for the effect of forest and other gradient were largely consistent regardless of the exact data generated. However, the results for altitude and distance to roads were more impacted by simulation uncertainty, with improvements only occurring for approximately 50% of simulated datasets.

*Quantification of variability introduced by background point selection*

In addition to stochasticity in the data generation process, there is also stochasticity in the selection of availability points for the citizen science models (both naïve and corrected). These points were selected randomly according to the corrected availability probabilities (as indicated from the observer model). The exact location of these points will therefore be different each time a random selection is conducted, in contrast to selecting points evening across a grid or mesh. As the estimated values of coefficients will be impacted by the exact location of the availability points (Warton and Shepherd 2010) some variability in results will be introduced. In order to provide an estimation of this variability repeated the availability point selection and model fitting 50 times for a single simulated dataset. Uncertainty in sampling weights was not assessed as all model fitting processes sampled points with the same probabilities. Improvement in coefficient estimates was recorded in the same way as for the assessment of uncertainty in the data generation process.

Figure S2 shows some of the variability in the improvement of coefficient estimates that is generated from stochasticity in the selection of availability points. For variables related to the observation process, the correction effect was robust, with > 95% of realisations of availability points generating an improvement in the estimated coefficient value. However, for other variables, such as altitude, which was not related to the observation process, improvements were approximately 50:50. This is not a surprising result and confirms that for a single dataset, our proposed model framework should be a robust way to correct coefficient estimates that are affected by an observation process.

*Cross-species observer model*

To explore the potential our method could provide, we attempted to use the observer model of one species to infer the suitability map of another species. For instance, we computed the observer model of roe deer and used it to correct for sampling biases in moose opportunistic observations.

Results indicate that this strategy could be useful for species with similar ecologies. For instance, using the observer model of roe deer to correct for moose opportunistic observation biases resulted in a suitability map very similar to when using the moose observer model (Figure A9). Even though not as correlated, using the wild reindeer observer model also could correct for sampling bias in moose opportunistic data. The same pattern holds true for roe deer, using the moose observer model resulted in a suitability map highly correlated to when using the roe deer observer model (Figure A10). Nevertheless, for wild reindeer, using the observer model of another species seems to hardly correct for sampling bias (Figure A11).

References:

Warton, D. I., & Shepherd, L. C. (2010). Poisson point process models solve the" pseudo-absence problem" for presence-only data in ecology. *The Annals of Applied Statistics*, 1383-1402.


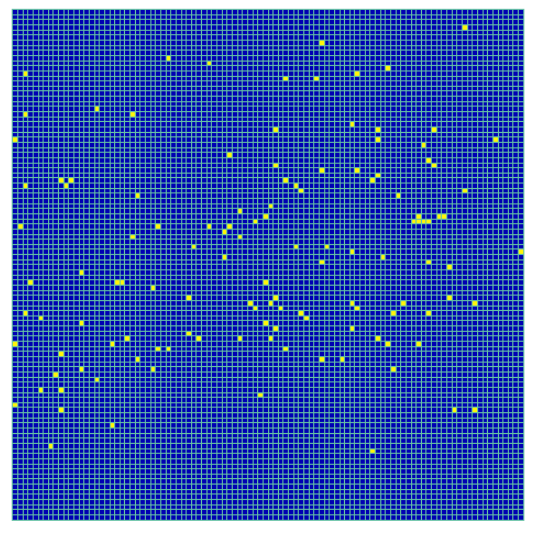

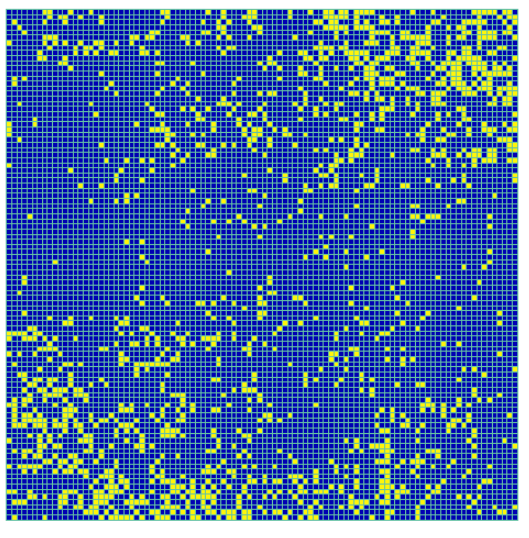


**Figure S1.** On the left, simulated citizen scientist presence in the simulated landscape and on the right, simulated species presence in the simulated landscape. Yellow dots represent presence while blue dots represent absence.


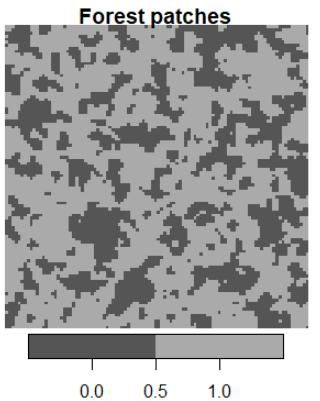

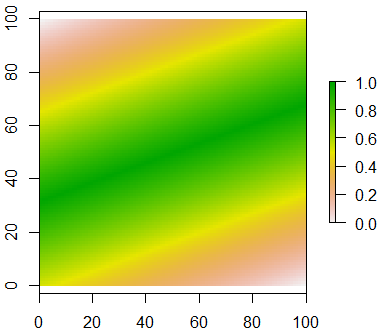

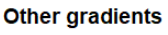

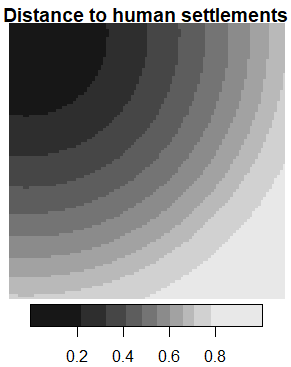

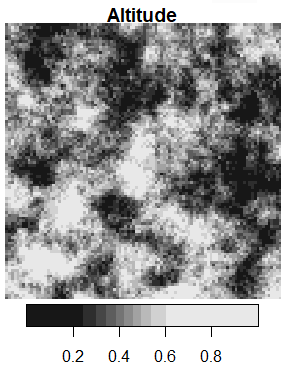

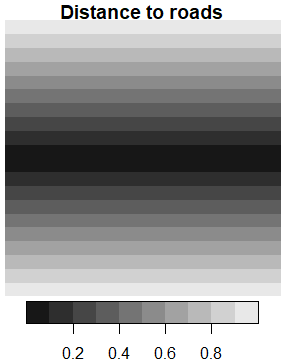

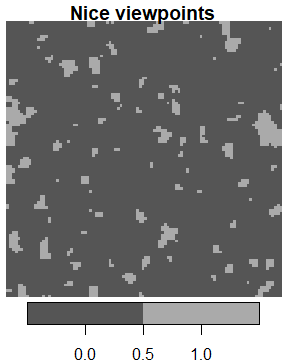


**Figure S2.** Simulated landscape variables

**
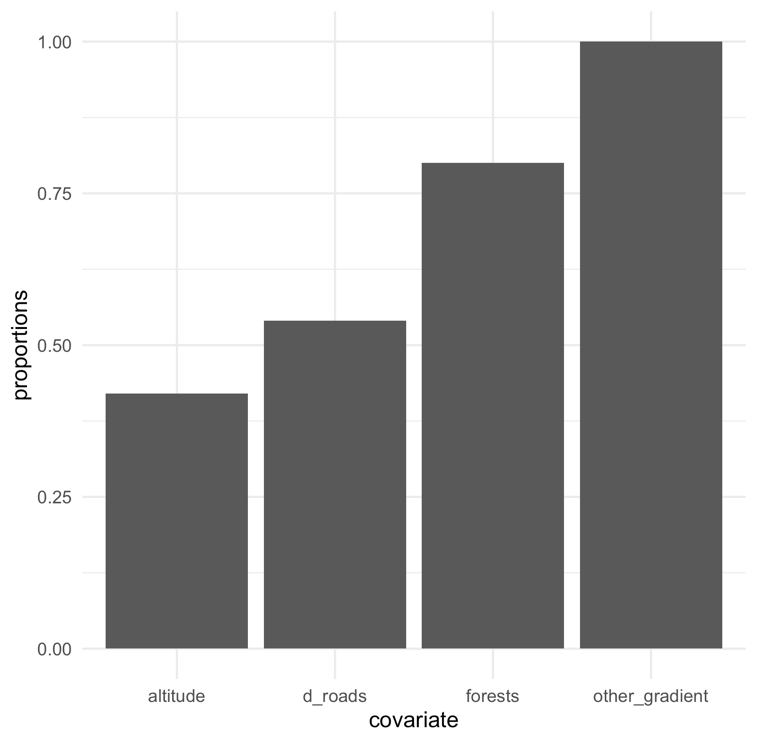
**

**Figure S3.** Proportion of times that the covariate estimate was improved by the corrected model. Results from 100 realisations of the data generation process.


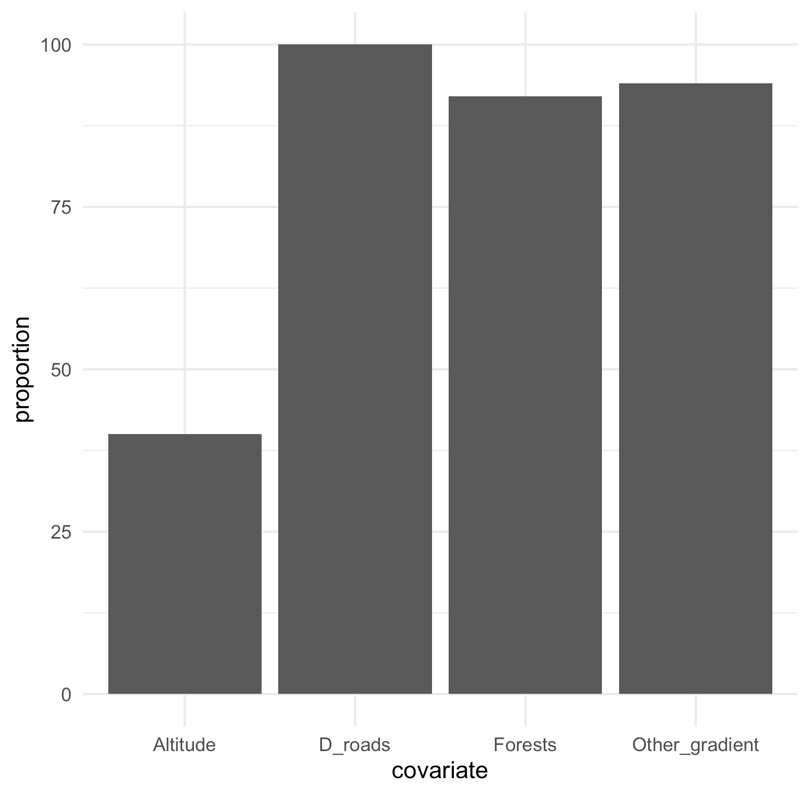


**Figure S4**. Proportion of times that the covariate estimate was improved by the corrected model. Results from 100 realisations of the background point selection process on a single dataset.


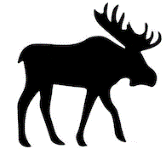

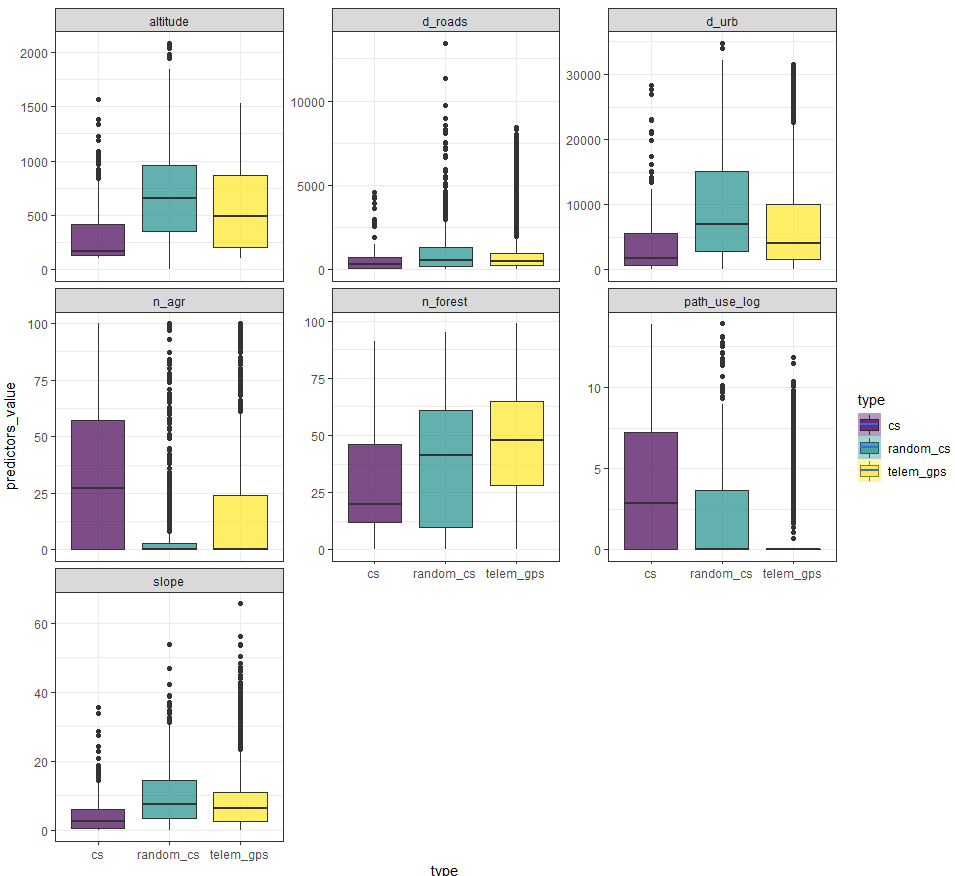


**Figure S5.** Histograms describing the distribution of the covariates used in the moose observer model and the moose RSF.


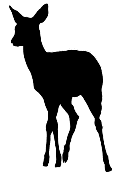

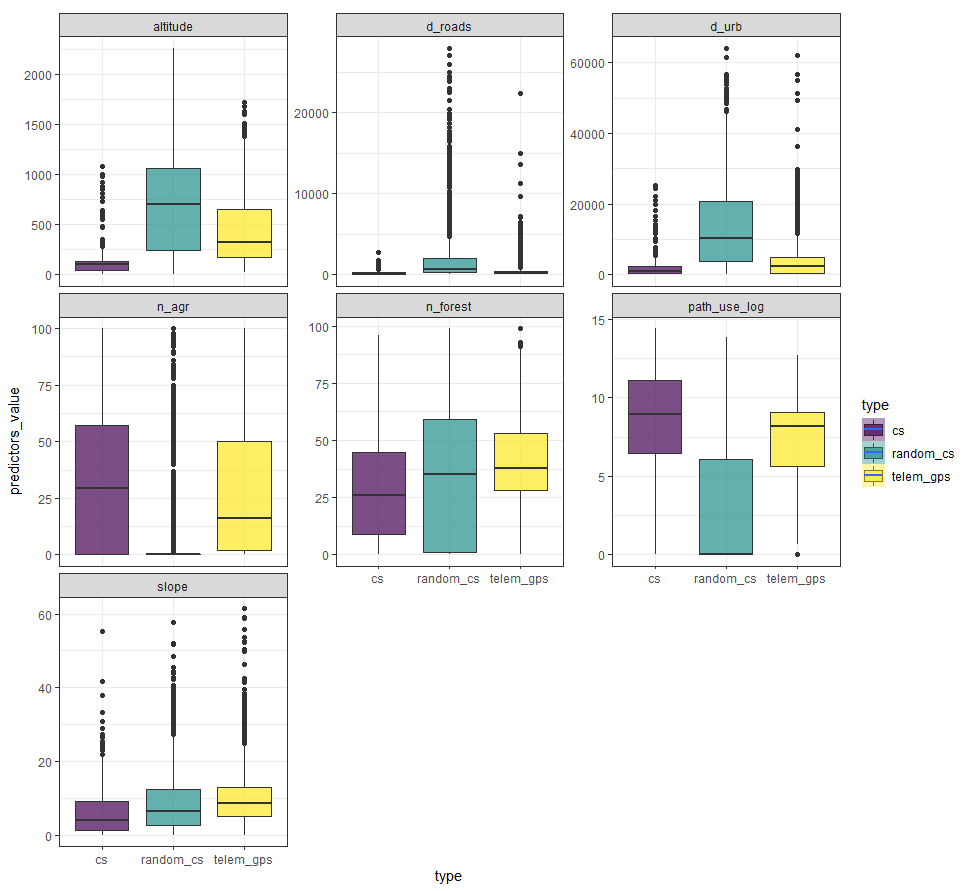


**Figure S6.** Histograms describing the distribution of the covariates used in the roe deer observer model and the roe deer RSF.


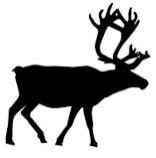

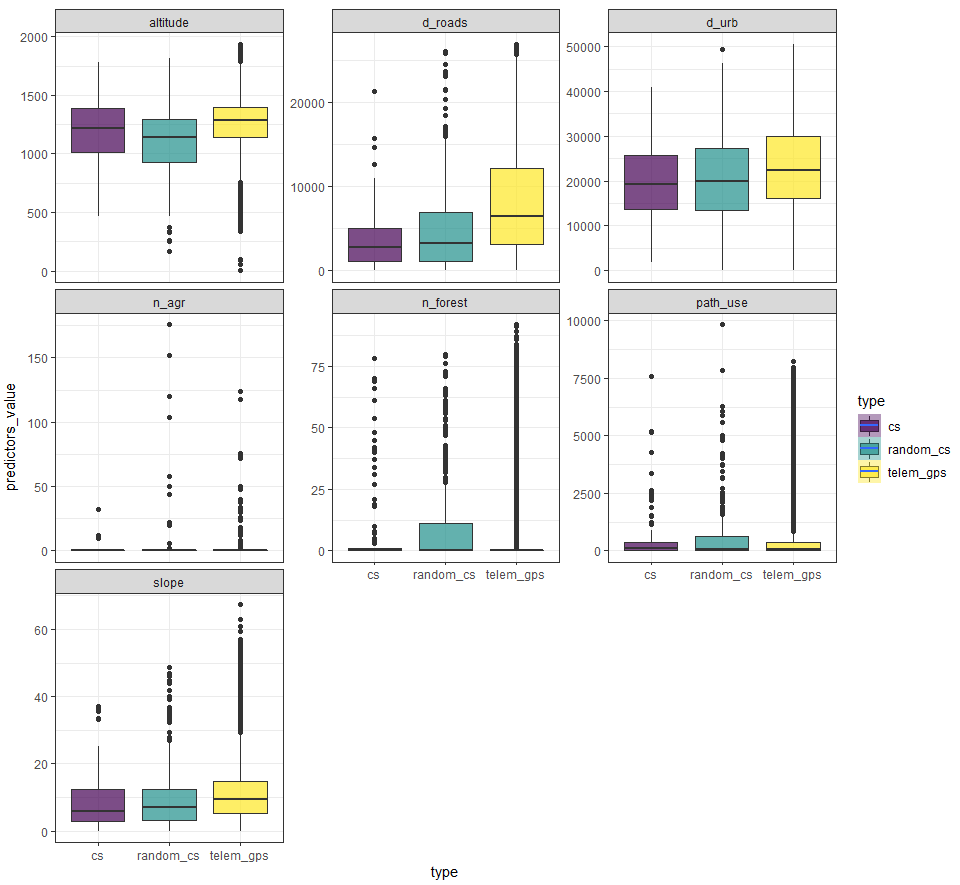


**Figure S7.** Histograms describing the distribution of the covariates used in the roe deer observer model and the roe deer RSF.


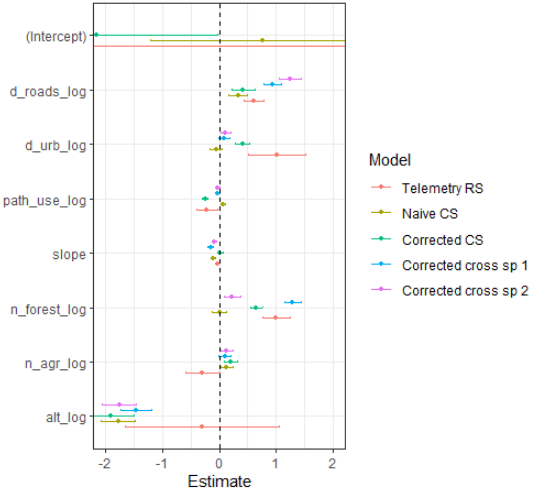

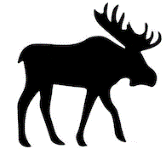


**Figure S8.** RSF parameter estimates for moose. In red are the parameter estimates obtained with the telemetry model, in yellow with the naïve CS model, in green with the corrected CS model, in blue with the corrected CS model using the roe deer observer model and in purple with corrected CS model using the wild reindeer observer model. Dots represent the mean parameter estimate and bars the 95 Credible intervals.


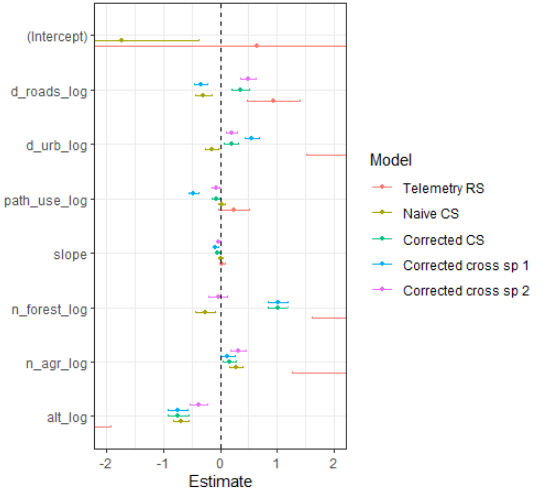

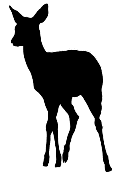


’

**Figure S9.** RSF parameter estimates for roe deer. In red are the parameter estimates obtained with the telemetry model, in yellow with the naïve CS model, in green with the corrected CS model, in blue with the corrected CS model using the moose observer model and in purple with corrected CS model using the wild reindeer observer model. Dots represent the mean parameter estimate and bars the 95 Credible intervals.


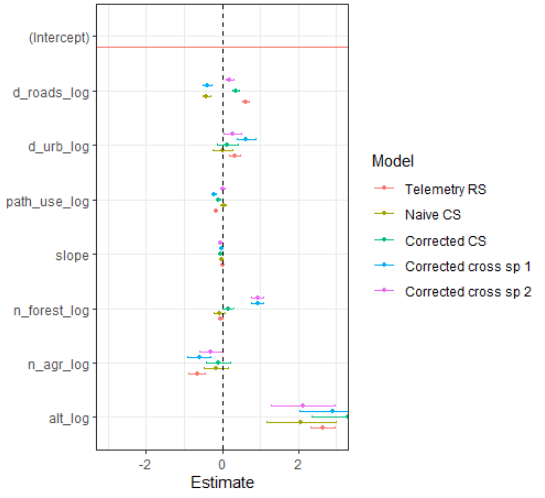

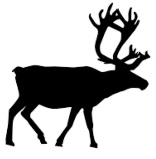


**Figure S10.** RSF parameter estimates for roe deer. In red are the parameter estimates obtained with the telemetry model, in yellow with the naïve CS model, in green with the corrected CS model, in blue with the corrected CS model using the moose observer model and in purple with corrected CS model using the roe deer observer model. Dots represent the mean parameter estimate and bars the 95 Credible intervals.


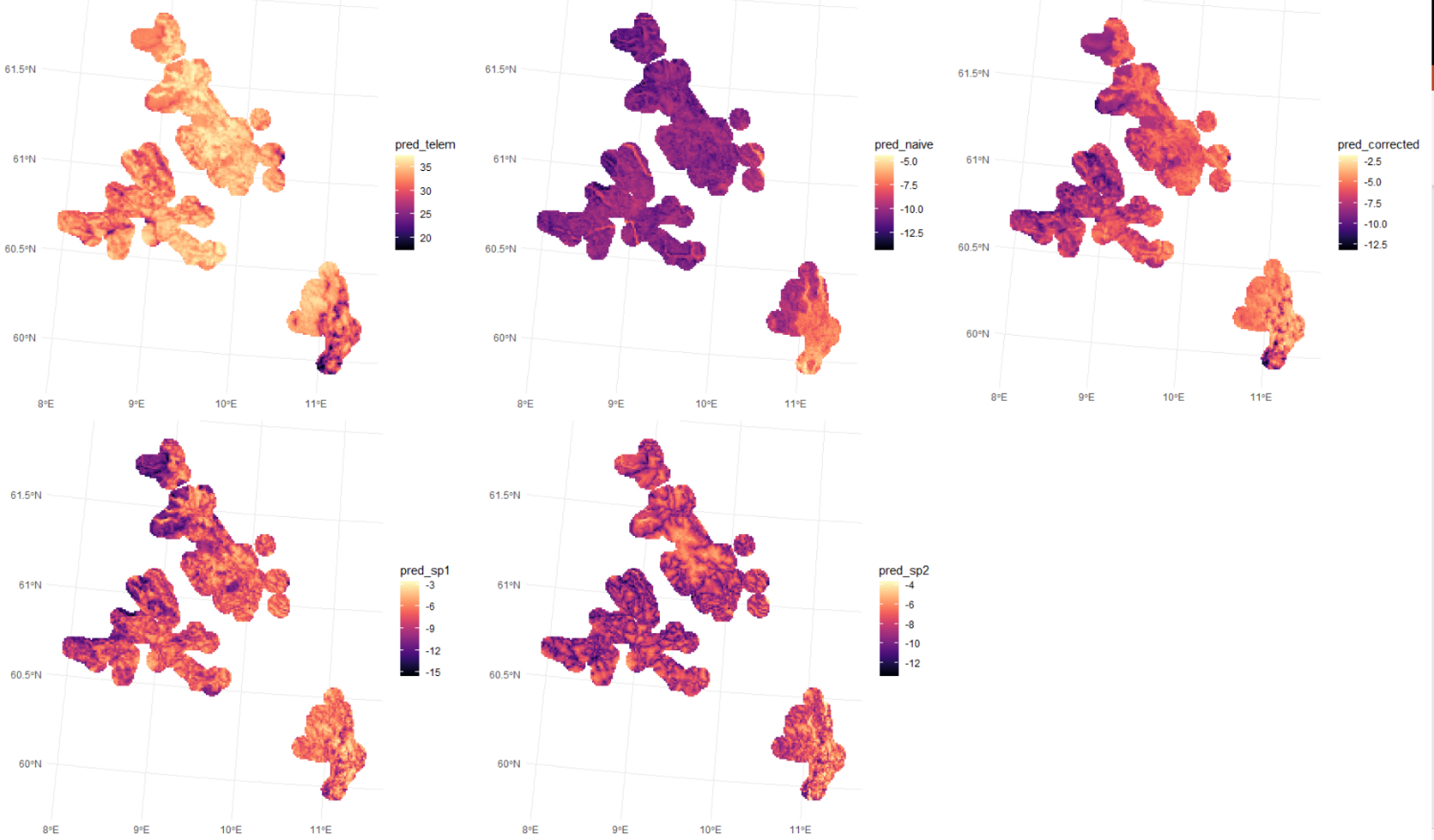

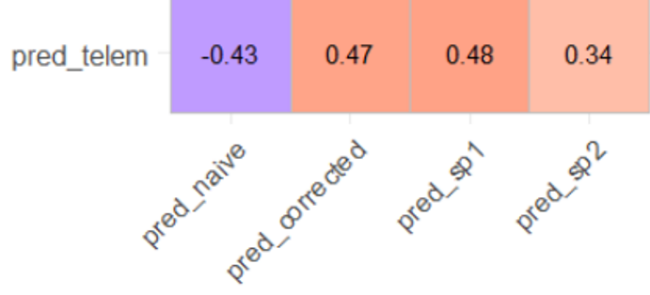


**a)**

**b)**

**c)**

**d)**

**e)**

**f)**


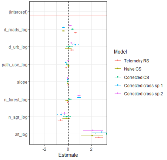


**Figure S11.** Moose suitability maps obtained using the mean coefficients of the a) telemetry model, b) naïve CS model, c) corrected CS model, d) corrected CS model using roe deer observer model and e) corrected CS model using wild reindeer observer model. On f) are the Pearson correlation coefficients between the habitat suitability maps obtained with the telemetry model and the ones obtained with the naïve CS model, the corrected CS model, the corrected CS model using roe deer observer model and the corrected CS model using wild reindeer observer model.


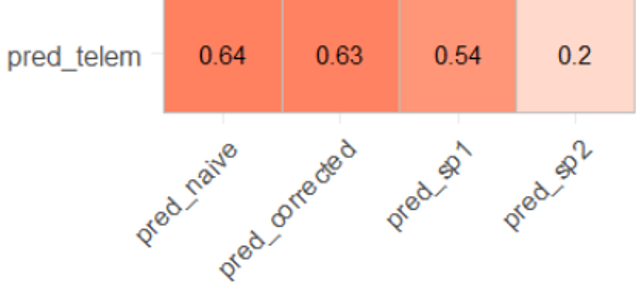

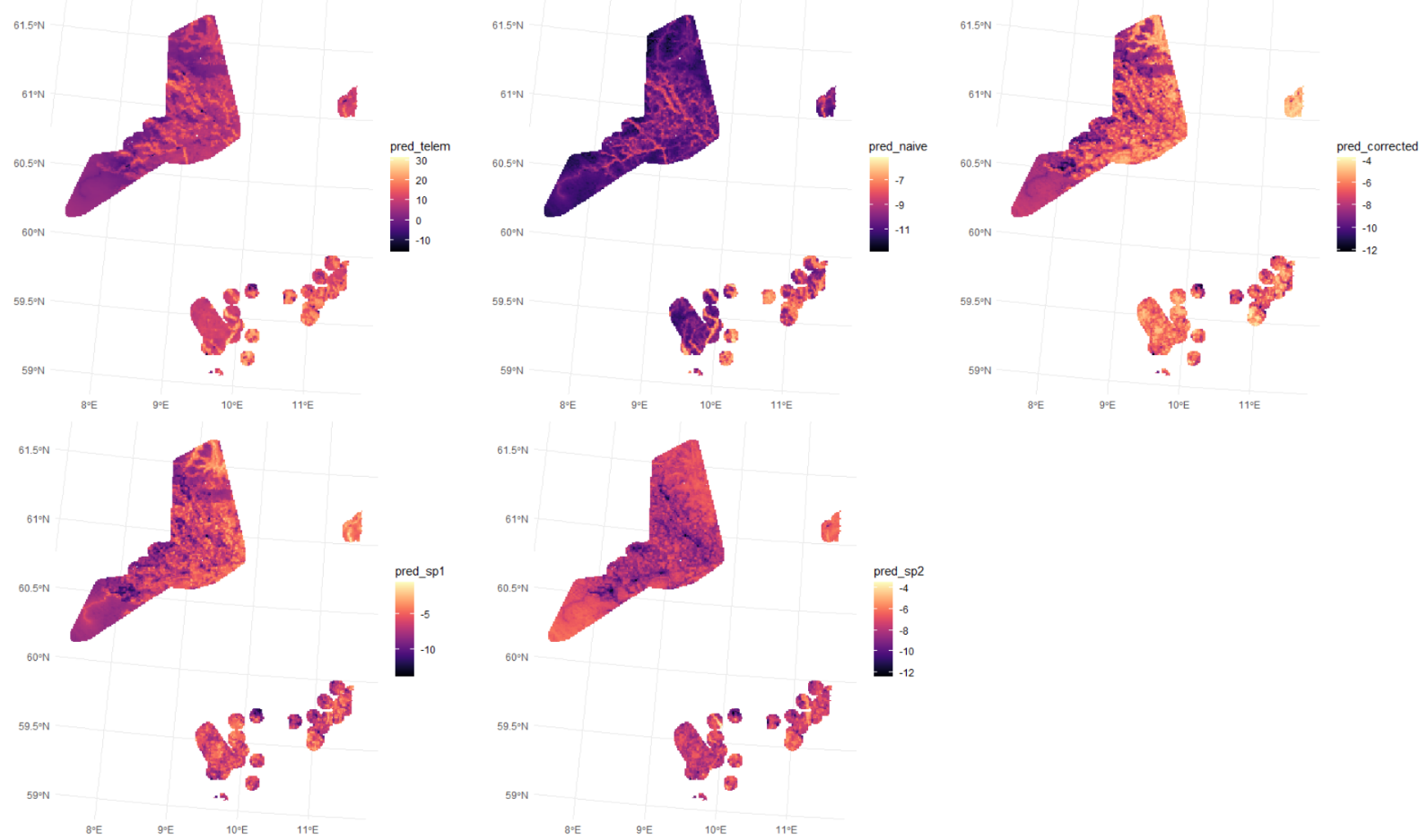


**a)**

**b)**

**c)**

**d)**

**e)**

**f)**


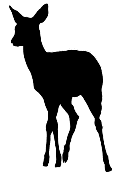


**Figure S12.** Roe deer suitability maps obtained using the mean coefficients of the a) telemetry model, b) naïve CS model, c) corrected CS model, d) corrected CS model using moose observer model and e) corrected CS model using wild reindeer observer model. On f) are the Pearson correlation coefficients between the habitat suitability maps obtained with the telemetry model and the ones obtained with the naïve CS model, the corrected CS model, the corrected CS model using moose observer model and the corrected CS model using wild reindeer observer model.


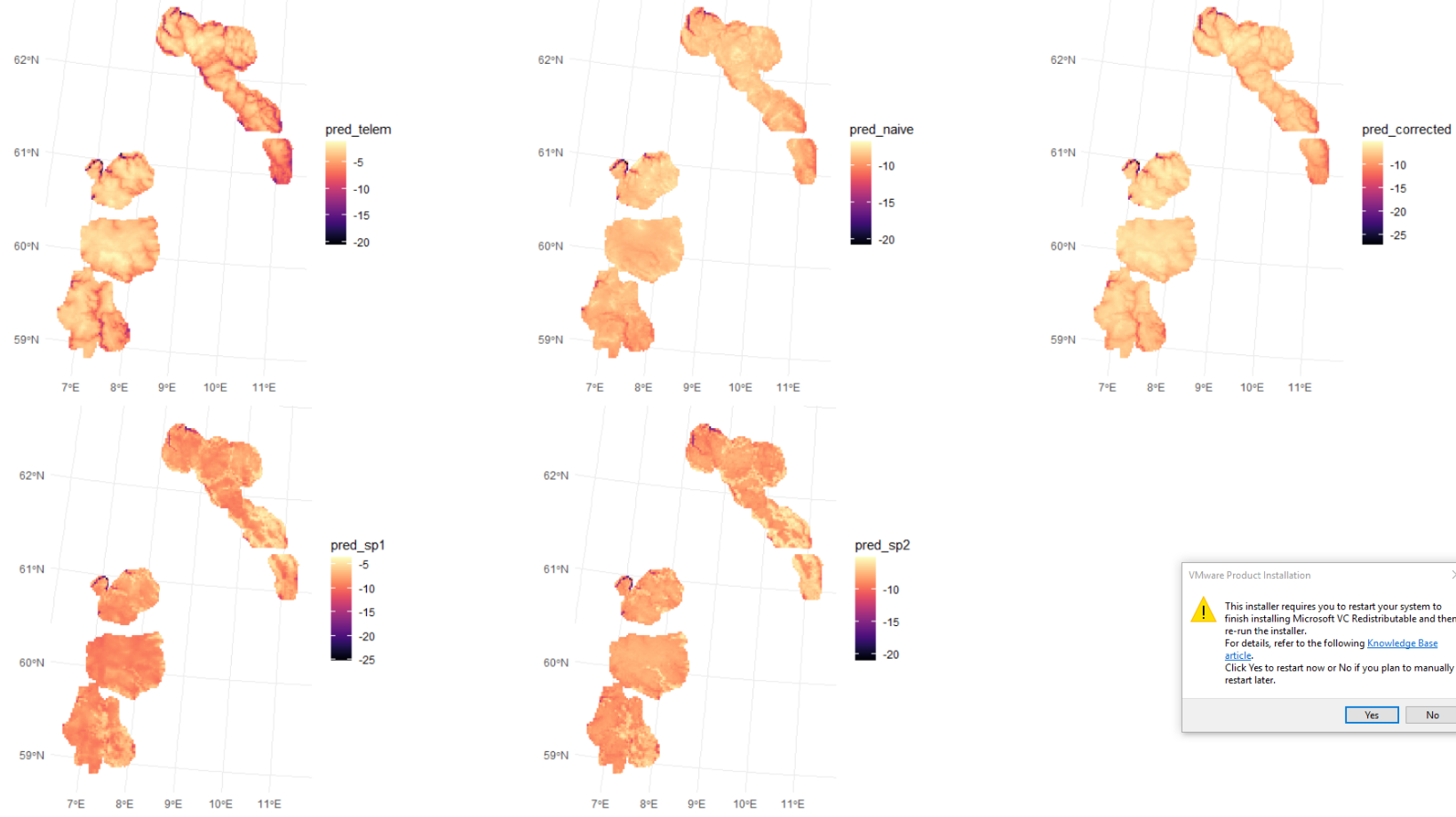

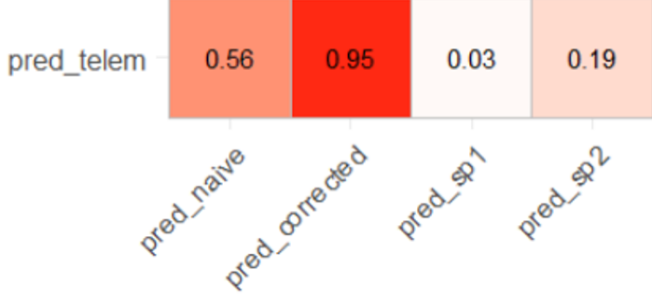


**a)**

**b)**

**c)**

**d)**

**e)**

**f)**


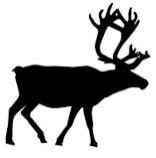


**Figure S13.** Wild reindeer suitability maps obtained using the mean coefficients of the a) telemetry model, b) naïve CS model, c) corrected CS model, d) corrected CS model using moose observer model and e) corrected CS model using wild reindeer observer model. On f) are the Pearson correlation coefficients between the habitat suitability maps obtained with the telemetry model and the ones obtained with the naïve CS model, the corrected CS model, the corrected CS model using moose observer model and the corrected CS model using roe deer observer model.
